# Genome‐wide analysis of single nucleotide variants allows for robust and accurate assessment of clonal derivation in cell lines used to produce biologics

**DOI:** 10.1002/bit.27534

**Published:** 2020-08-24

**Authors:** Alexandre Kuhn, Valérie Le Fourn, Igor Fisch, Nicolas Mermod

**Affiliations:** ^1^ Department of Fundamental Microbiology, Institute of Biotechnology University of Lausanne Switzerland; ^2^ Selexis SA Geneva Switzerland

**Keywords:** biologic, cell line development, clonal derivation, genomics, high‐throughput sequencing, monoclonality

## Abstract

A clonally derived (or “monoclonal”) cell line is a cell population derived from a single progenitor cell. Clonally derived cell lines are required for many biotechnological applications. For instance, recombinant mammalian cells used to produce therapeutic proteins are expected by regulatory authorities to be clonally derived. Assurance of clonal derivation (or “clonality”) is usually obtained from the characterization of the procedure used for cell cloning, for instance by assessing the success rate of single‐cell sorting but not by assessing the cell line itself. We have developed a method to assess clonal derivation directly from the genetic makeup of cells. The genomic test of clonality is based on whole‐genome sequencing and statistical analysis of single nucleotide variants. This approach quantifies the clonal fractions present in nonclonal samples and it provides a measure of the probability that a cell line is derived from a single cell. Upon experimental validation of the test, we show that it is highly accurate and that it can robustly detect minor clonal fractions of as little as 1% of the cell population. Moreover, we find that it is applicable to various cell line development protocols. This approach can simplify development protocols and shorten timelines while ensuring clonal derivation with high confidence.

## Graphical abstract

In many biotechnological applications, the assurance of clonal derivation (“monoclonality”) is usually obtained from the characterization of the procedure used for cell cloning. Kuhn et al. have developed a method to assess clonal derivation directly from the cells’ genetic material. They show that their “Genomic Test of Clonality” is highly accurate and that it can be integrated into various cell line development protocols. This approach has the potential to simplify development protocols while ensuring clonal derivation with high confidence.

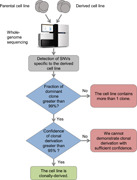

## 1 INTRODUCTION

Recombinant cell lines used to produce biologics for therapeutic use should be clonally derived (i.e., “monoclonal”), that is they should derive from a single progenitor cell. Specifically, regulatory authorities expect that master cell banks (i.e., the cell line used for manufacturing) be thoroughly documented for their clonal derivation. The rationale for this requirement is that such a cell bank is genetically more homogeneous, which can improve the consistency and robustness of recombinant protein. In contrast, if several clonal populations are present within the cell line, potential changes in manufacturing conditions could put selective pressure on the cells, possibly resulting in modified clonal composition and variations or heterogeneity of the final product (Welch & Arden, 2019).

Most efforts geared at ensuring clonal derivation (or “monoclonality”) have relied on characterizing single‐cell cloning procedures. Hence, from the classical limiting dilution method to the recent development of microfluidic chips to sort single cells individually, every new technology has improved the effectiveness of single‐cell cloning. In contrast, direct assessment of clonal derivation based on the analysis of the recombinant cell line itself has witnessed few improvements. Indeed, until recently, molecular genetic methods that could help to assess clonality directly were very laborious (e.g. analyzing transgene integration sites) or had poor resolution (e.g. Southern blots). The advent of high‐throughput sequencing, however, has opened new possibilities.

High‐throughput DNA sequencing‐based methods can now readily identify transgene integration sites. When transgene integration is random, integration sites can be used as a unique genetic feature of the cell line, that is as a clonal signature. The clonal derivation of a cell line can thus be established by verifying that the specific integration site is homogeneously present in the population. For instance, Aebischer‐Gumy, Moretti, Little, and Bertschinger (2018) have assessed cell line clonality by generating individual subclones and by verifying that a large number of these subclones contained the same specific genome‐transgene junction.

Over the last years, however, it has become increasingly clear that even clonally derived cell lines can gradually become genetically heterogeneous. Indeed, several studies have shown that subclones from the same cell line can display different phenotypic behaviors (Ko et al., 2018; Patel et al., 2018; Tharmalingam et al., 2018). In addition, clonally derived cell lines have been shown to display genetic evolution affecting their genome at various scales (from the accumulation of point mutations to chromosomal rearrangements), as well as epigenetic changes (Feichtinger et al., 2016; Vcelar et al., 2018). However, a systematic understanding of how genetic and epigenetic changes relate to phenotypic characteristics of a cell line is lacking.

Here, we show that non‐clonal cell lines can be efficiently detected based on the genome‐wide analysis of its single nucleotide variants (SNVs). Building on this principle, we developed and validated a formal statistical procedure for testing clonal derivation named genomic test of clonality (GTC). We derived the minimal requirements needed to ensure the high sensitivity of the test. We also show that this procedure is very robust and that it does not depend on details of either the sequencing technology or the bioinformatic analysis used for the identification of SNVs for instance. Importantly, we demonstrate that it can be efficiently integrated in the context of commercial cell line development, even in the case of procedures involving multiple successive subcloning steps.

## 2 MATERIALS AND METHODS

### 2.1 Cell lines, whole‐genome sequencing and detection of SNVs

All cell lines used here were derived from the Chinese hamster ovary (CHO) host cell line HCB‐2 (Selexis SA), except for the cell lines SG and FG that were derived from the host cell line HCB‐1 (Selexis SA). Both HCB‐1 and HCB‐2 lines were obtained from the CHO‐M (Selexis SA) cell line, originally derived from a CHO‐K1 line. Construction of expression vectors, transfection conditions, and cell culture conditions were previously described (Grandjean et al., 2011; Le Fourn, Girod, Buceta, Regamey, & Mermod, 2014). The details of sample preparation for all experiments presented here are provided in the Supporting Information.

Sample‐specific SNVs are SNVs that can be detected in the tested cell line (e.g. with a frequency >0.05, i.e., 5%) but that have very low frequency in the parental population. We performed whole‐genome sequencing using Illumina technology and detected sample‐specific SNVs using standard bioinformatics methods (see the Supporting Information), including the R/Bioconductor package VariantTools (Lawrence, Degenhardt, & Gentleman, 2019). Restricting to SNVs that are very rare in the parental population allows us to measure clonal fractions in the tested population. Specifically, it ensures that if there are two progenitor cells (in the event of failed cell cloning), the probability that they will each contain a different, specific set of SNVs is very high (see Section 2.2.1). Specifically, we selected all SNVs that were present in the tested cell line and undetected in the parental cell population (i.e., no variant allele observed). Considering the high sequencing depth used for HCB‐2, this corresponds to SNVs with variant allele frequencies <0.05. Moreover, we restricted sample‐specific SNVs located in regions that were present in a single copy in the genomes of both the tested cell population and the parental cell line. As fixation of such SNVs results in allele frequencies of one (whereas fixation of an SNV located in a 2‐copy region results in an allele frequency of 0.5), this facilitates the estimation of clonal fractions from the parametric model (see Section 2.2.2).

### 2.2 Genomic test of clonality

#### 2.2.1 Parametric model

Let us consider SNVs that are specifically detected in the cell population tested for clonal derivation. Each SNV is characterized by the coverage **c**_**d**_ (i.e., the total number of sequencing reads covering the genomic position) and the number of sequencing reads carrying the variant allele **a**_**d**_ (also referred to as the alternative allele count). The frequency of a variant is defined as the ratio **a**_**d**_/**c**_**d**_. We assume that each SNV is specific to a given clone, that is a given SNV cannot be present in two clonal subpopulations in the case of a clonal mixture (see “Verification of model specification” in the Supporting Information for the assessment of this assumption). For a given SNV in a 1‐copy region of the genome, **a**_**d**_ is modeled as a variable from a Binomial distribution with parameters **c**_**d**_ (number of trials) and **f** (probability, corresponding to the fraction of the clone in the cell population). In other words, **a**_**d**_ is distributed as the number of “successes” (i.e., reads carrying the variant allele) in a series of **c**_**d**_ independent experiments (i.e., the total number of reads covering that genomic location), each with probability **f** to “succeed” (i.e., the read originates from the clonal population carrying the variant allele). In addition, the coverage **c**_**d**_ varies across SNVs and it can be modeled as a variable from a Poisson distribution (Lander & Waterman, 1988) with parameter **C** (true average coverage). Thus, considering the set of SNVs corresponding to a given clone, the alternative allele count **a**_**d**_ can be modeled as a Binomial distribution (i.e., the probability of observing the alternative allele count for a fixed coverage) compounded by the Poisson‐distributed coverage (see e.g. Ocerin & Pérez, 2002). This compound distribution is another Poisson distribution with parameter **C** * **f** (true average coverage times clonal fraction). Finally, if we assume that the cell population is composed of two clones, we can model the variant allele count for all SNVs in the population as a mixture of two Poisson distributions (with parameters **C** * **f** and **C** * **(1 − f)**, respectively, see Figure S1a).

#### 2.2.2 Parameter estimation

We aim to estimate**f** from the parametric mixture model described above. In the present case, directly fitting a mixture of Poisson distributions is unpractical, however, because the alternative allele count distribution is usually left truncated (at the minimal number of reads set to detect SNVs reliably, e.g. here four reads) or contaminated by artefactual SNVs. To circumvent this problem, we developed a 2‐step procedure: We obtain an initial estimate of **f** that allows for the detection of balanced to moderately unbalanced (e.g. **f** = 0.5–0.8) clonal mixtures without making any assumption. Based on this initial measure, we next determined a second, very accurate measure of **f** for mixtures with larger major clonal fractions (and correspondingly smaller minor fractions). This refined measure of **f** provides the high sensitivity needed to detect highly unbalanced samples.

The initial estimate of **f** is obtained by fitting the upper component of the Poisson mixture only. Specifically, we fit a single, truncated Poisson distribution, whose truncation level is given by half the mean coverage (i.e., **c̅**_**d**_/2), allowing to estimate **C** * **f** (Figure S1b). The fraction of the majority clone **f** is given by **f**_**pois**_ = (**C** * **f**)/**c̅**_**d**_. This initial measure of **f** allows us to efficiently and safely reject monoclonality in the cases of clonal mixtures that are not too unbalanced. When clonal ratios are greater than approximately 80/20, however, we can obtain a more accurate measure of **f** by identifying SNVs from the majority clone and fitting them specifically, hence removing the mixture problem. We define the two Poisson component distributions as “separable” when their overlap is small enough such that the probability of making an error when assigning each allele count (i.e., SNV) to a clonal subpopulation has a fixed upper bound (specifically, if there is a count value such that at least 95% of counts from the majority clone are strictly greater and at most 5% of counts from the minority clone are strictly smaller, see Figure S1a). Separability depends on SNV coverage: the higher the coverage, the greater the separability (conversely, if coverage is low, e.g. <5, even two clones with a very unbalanced clonal ratio, e.g. 90/10, will not be separable). If the initial Poisson‐based measure **f**_**pois**_ is greater than the minimal fraction ensuring separability (for the observed average coverage **c̅**_**d**_) (Figure S1c), we identify SNVs from the majority clone and use them to calculate the more accurate, Binomial‐based measure of **f**. For a typical average coverage of 13, the minimum (true) **f** allowing separability is 0.81.

SNVs from the majority clone are identified as follows (Figure S1d): for each SNV coverage value, we determine the count value such that when most of the allele counts (i.e., at least 99%) of the distribution from the majority clone (modelled as Binomial with parameters **c**_**d**_ and **f**_**pois**_) are included, the probability of having an allele count of the distribution of the minority clone (modelled as Binomial with parameters ***c***_**d**_ and 1 − **f**_**pois**_) above this count value is upper bounded (at most 5%). Thus, SNVs with counts above this boundary are confidently assigned to the majority clone (Figure S1d). When restricting to SNVs identified as originating from the majority clone, we obtain a more accurate measure of **f** as follows: as the probability parameter of these Binomial variables is identical, their sum ($\sum a_{d}$) follows a Binomial distribution with parameters $\sum c_{d}$ and **f**, and **f** is thus given by **f**_**binom**_ = $\sum a_{d}/\sum c_{d}$. We finally used the Agresti‐Coull method to calculate a confidence interval for this binomial fraction estimate. A sample is called (clonally) pure only if the lower limit of the Agresti‐Coull confidence interval for **f**_**binom**_ is >0.99 (see “Operational threshold for clonal purity” in the Supporting Information). All statistical procedures were implemented using the R software (R Core Team, 2020).

#### 2.2.3 *p‐*value for clonal derivation

For a cell population that is deemed clonally pure, we can obtain a *p*‐value corresponding to the hypothesis that it is derived from a single progenitor cell (i.e., clonal derivation, or monoclonality). For the sake of illustration, let us first consider a single individual SNV that is rare in the parental population and becomes fixed upon cell cloning. If single‐cell cloning fails and there are two progenitor cells instead of 1, we will wrongly call the population clonally derived if, and only if, the second cell bears the same SNV. If we assume that the two progenitor cells are randomly drawn from the population, the probability that the second cell bears the same SNV is given by its population frequency. This frequency thus formally corresponds to a statistical *p*‐value for clonal derivation (i.e., the probability of making an error if we accept the null hypothesis that the population is clonally derived).

If we now consider more than 1 fixed SNV in the derived population, the probability that the exact same set of SNVs is carried by the second progenitor cell is bound to be lower than the probability obtained with a single SNV, such that the confidence of clonal derivation is higher. Formally, the *p*‐value corresponding to the smallest SNV frequency observed in the parental population corresponds to the upper bound (i.e., conservative estimate) of the true *p*‐value.

In practice, the measurement of SNV frequency via standard sequencing library preparation and sequencing has a limited resolution. Owing to the intrinsic per‐base error in the current best standard sequencing technologies, the limit of rare variant detection is (conservatively) estimated at 0.05. This limit can be reached with a sequencing depth >50×–100× and higher sequencing depths do not improve the sensitivity of detection. Here, we thus performed deep sequencing of the host cell population (i.e., 150× for HCB‐2) and considered that if we did not observe a single alternative allele in the parental population (i.e., for an SNV fixed in the derived population), it had a frequency <0.05, yielding a *p*‐value for clonal derivation <0.05. Importantly, we have shown that SNVs fixed in derived cell lines are at least ×10 rarer than this (i.e., <0.005, see “Verification of model specification” in the Supporting Information). This implies again that in the present application of GTC, the true *p*‐value for clonal derivation is significantly less than the upper bound of 0.05.

## 3 RESULTS

### 3.1 Characterization of the genetic diversity in parental host cells and in clonally derived cell lines

**Figure 1:**
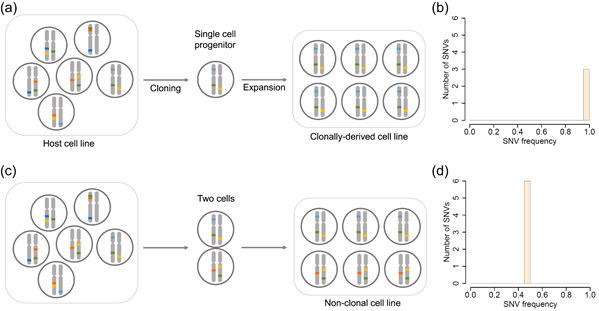
Schematic representation of genetic diversity in a host cell line and derived cell lines. (a) Cells in the host cell line contain a variety of rare mutations (specifically single nucleotide variants (SNVs), represented as color ticks on the chromosomes). The genome is depicted as a single pair of chromosomes for simplicity. Upon cell cloning SNVs harbored by the progenitor cell (and represented by the blue, green, and yellow ticks) are inherited by all daughter cells and thus become fixed in the clonally derived cell line. (b) Population frequency of SNVs in the clonally derived cell line (represented in panel a) showing that three SNVs are detected with a population frequency of 1 (i.e., present in all cells). (c) Non‐monoclonal cell line derived from two progenitor cells. Each progenitor cell contains specific SNVs and the derived cell line is composed of two subpopulations that are each derived from one of the progenitor cells. (d) Population frequency of SNVs in the derived non‐monoclonal cell line (represented in panel c) showing six SNVs each with a population frequency of 0.5 (i.e., present in half of the cells) [Color figure can be viewed at wileyonlinelibrary.com]

In the process of producing a new cell line for recombinant protein production, a cell population (i.e., the “host” or “parental” population) is transfected with a vector directing the expression of the recombinant protein. A single cell is isolated from that population and gives rise to the new cell population (i.e., the derived cell line) upon cell divisions (Figure 1a). If there is abundant genetic diversity in the host cell line, the single‐cell cloning step is expected to result in a dramatic decrease in the genetic diversity of the derived cell line as compared to the host cell line: mutations contained in the progenitor cell will be inherited by daughter cells, whereas other genetic variants present in the host cell line will be absent from the derived cell line. In contrast, if cell cloning fails and two progenitor cells lead to a non‐monoclonal cell line, the derived population will contain two cell subpopulations that each inherited from the genetic variants contained in the corresponding progenitor cell (Figure 1c).

Here, we specifically consider the genetic diversity provided by SNVs but the argument holds true for other types of mutations as well. The difference in the genetic makeup of a population originating from one or two progenitor cells is reflected in the SNV frequency spectrum of the derived population. In the case of a single progenitor cell, all SNVs are fixed and thus have a population frequency of 1 (Figure 1b) whereas if there are two progenitor cells and each cell bears specific SNVs, the SNVs in the derived population are present in only half of the cells and thus have a frequency of 0.5 (Figure 1d). We will show that this difference can provide the basis for a formal test of clonality.

To verify our assumptions, we first characterized SNVs in a standard host cell line (CHO‐K1 derivative) that is routinely used to generate cell lines for recombinant protein production, as well as in a clonally derived cell line. Both samples were subjected to whole‐genome sequencing and SNVs were detected using standard bioinformatics methods. The vast majority of SNVs detected in the host cell line were low‐frequency (<0.2, i.e., 20%) mutations whereas the derived cell line indeed contained many newly fixed mutations (Figure S2), as anticipated.

### 3.2 Cell lines derived from one or from two progenitor cells display different SNV frequency spectra

**Figure 2:**
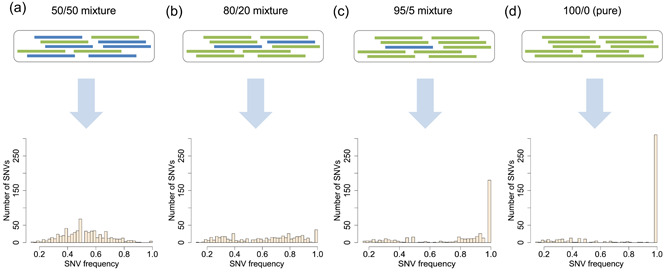
Simulation of mixed clonal populations and analysis of their single nucleotide variant (SNV) frequency spectra. Sequencing reads originating from whole‐genome sequencing of two clonally derived cell lines (represented by blue and green rectangles) were combined to simulate clonal mixtures presenting varying ratios of the two cell lines. Each mixture was subjected to SNV analysis and the corresponding SNV frequency spectrum is represented at the bottom. The ratio of the two cell lines in each artificial sample is as follows (percentage of green cell line/percentage of blue cell line): (a) 50/50, (b) 80/20, (c) 95/5, (d) 100/0 (monoclonal population) [Color figure can be viewed at wileyonlinelibrary.com]

We, then, explored the detection of clonality based on the analysis of SNV frequencies using clonal mixtures generated in silico. Specifically, we subjected two clonally derived cell lines to whole‐genome sequencing and generated mixed samples by combining their sequencing data. To obtain a sample mimicking a balanced (50/50) clonal mixture (corresponding to the hypothetical case illustrated in Figure 1c,d), we pooled equal proportions of reads from each of the two clonally derived cell lines (Figure 2a). The analysis of this mixed sample revealed that sample‐specific SNVs had frequencies centered around 0.5, as anticipated.

We repeated the mixing procedure to generate samples mimicking clonal mixtures of varying ratios (80/20 and 95/5 in Figure 2b and 2c, respectively). Upon detection of SNVs, we observed fewer SNVs with frequency around 0.5 and relatively more SNVs with lower (<0.5) or higher (>0.5) frequencies. This reflects the underlying presence of two SNV clusters: The lower cluster originates from SNVs fixed in the cell line representing the minor clonal fraction, whereas the higher SNV cluster contains SNVs fixed in the cell line representing the majority clone (see also Figure S3). Moreover, the greater the fraction of the majority clone, the greater the number of high‐frequency SNVs. In conclusion, the analysis of SNVs can reveal the presence of two clonal populations even when unbalanced and their frequencies provide quantitative information on clonal fractions.

### 3.3 The genomic test of clonality (GTC) has high accuracy and sensitivity

Building on the statistical analysis of SNVs, we developed an accurate measure of the clonal fractions (potentially) present in a cell population and derived a measure of the probability that the cell line is clonally derived (i.e., *p*‐value for clonal derivation, or “monoclonality”) (see Section 2 and Figure S4). The procedure relies on the whole genome sequencing of the cell line and its corresponding host cell population. Subsequent bioinformatic analysis allows us to identify SNVs that are specific to the derived cell line (i.e., SNVs that have very low frequency in the parental population). The detected SNVs are then used to run GTC, which involves two steps: First, we fit a parametric model that yields measures of clonal fractions. If the cell population is deemed pure (clonal homogeneity), the second step calculates a *p*‐value for clonal derivation. Specifically, we test the hypothesis that the cell population is derived from a single progenitor cell. In the terms of statistical inference, the *p*‐value thus is the probability that the cell population is not clonally derived and actually derives from two progenitor cells bearing the same SNVs (indeed, if the two progenitor cells do not carry the same SNVs, clonal heterogeneity is detected in the first step of GTC).

**Figure 3:**
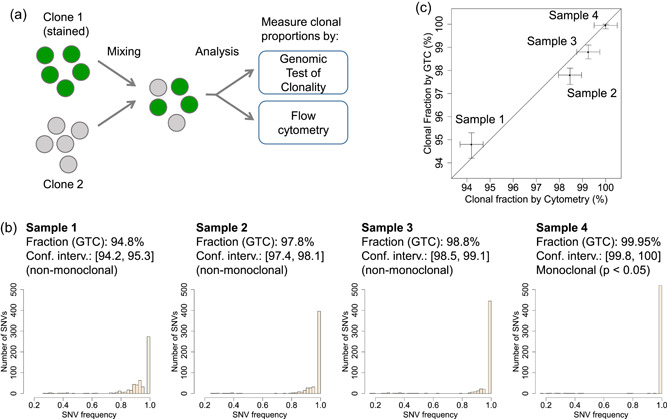
Experimental validation of clonal fractions measured by the genomic test of clonality (GTC). (a) We experimentally mixed two clonally derived cell lines in varying cellular proportions and applied GTC to measure clonal fractions. Cells from one cell line were stained beforehand and the actual cellular composition in each mixture was measured by flow cytometry as well. (b) Single nucleotide variant (SNV) frequency spectra, clonal fractions, corresponding confidence intervals, and *p*value for clonal derivation provided by GTC analysis for three highly unbalanced samples and a pure sample. GTC deemed Samples 1–3 as clonally heterogeneous (non‐monoclonal) and Sample 4 as clonally pure and clonally derived (*p* < .05). (c) Comparison of clonal fractions measured by GTC and by flow cytometry. The bars represent confidence intervals [Color figure can be viewed at wileyonlinelibrary.com]

We tested the accuracy of the clonal fractions measured by GTC and the sensitivity of the method using a cellular mixing experiment. In short, cells from two clonally derived cell lines were mixed in various ratios (Figure 3a) to determine what was the lowest minor clonal fraction that GTC could robustly detect. We analyzed the following mixed populations (percentage of major clone/percentage of minor clone): 95/5, 98/2, 99/1, and 100/0 (i.e., a pure clone). To independently verify the actual clonal proportions in the mixed samples, one of the two clonal populations was stained beforehand so that sample composition could be independently measured using flow cytometry. Finally, each mixed sample was subjected to whole‐genome sequencing and analyzed using GTC in a blinded fashion. It is important to highlight that the identity of SNVs fixed in each of the two clones was not known a priori to GTC.

Analysis of the mixed samples showed that SNVs yield a strong and robust signal for clonality assessment (Figure 3b). As the fraction of the major clone increased from 95% (Sample 1) to 100% (Sample 4), the distribution of SNV frequencies gradually shifted to the right towards 1. Based on these SNVs, GTC could infer measures of clonal fractions (along with tight confidence intervals, as shown at the top of each barplot in Figure 3b). Samples 1–3 were deemed clonally heterogeneous and thus non‐monoclonal. Sample 4 presented mostly fixed SNVs and was deemed (clonally) pure and clonally derived (*p* < .05). Comparison with parallel flow cytometry‐based measurements showed that the clonal fractions obtained with GTC were at least as accurate (Figure 3c). Notably, GTC could differentiate between samples composed of 99% of a clone (Sample 3) and the corresponding pure sample (Sample 4), demonstrating that it can robustly detect minor fractions of contaminating cells as low as 1%.

### 3.4 Validation of GTC in the context of cell line development

**Figure 4:**
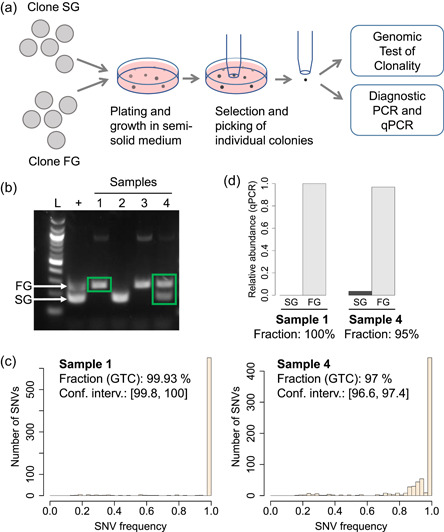
Validation of genomic test of clonality (GTC) in the context of cell line development using an automated cloning and imaging system. (a) A cell suspension composed of two clonally derived cell lines (named FG and SG) was plated in semi‐solid medium. Ten days later, individual colonies were picked according to standard imaging criteria. Colonies were screened by polymerase chain reaction (PCR) to identify clonal and non‐clonal colonies. Selected clonal and non‐clonal samples were subjected to GTC and quantitative PCR (qPCR). (b) Result of the PCR screen. Gel electrophoresis analysis of four samples showing FG‐specific amplicon only (Samples 1 and 3), SG‐specific amplicon only (Sample 2) or both amplicons simultaneously (Sample 4), hence revealing a colony composed of both clones. Green rectangles highlight samples displayed in panel c. (c) Single nucleotide variant (SNV) frequency spectra and results of GTC applied to Samples 1 and 4. Sample 1 was deemed clonally pure. It could not be called clonally derived because the sequencing depth of the parental cell line HCB‐1 was insufficient to reach a significance threshold of 0.05. Sample 4 was found to be clonally heterogeneous (and thus non‐monoclonal). (d) Fractions of the FG and SG clones measured by qPCR in Samples 1 and 4, in line with the clonal fractions measured by GTC [Color figure can be viewed at wileyonlinelibrary.com]

We asked if GTC could be applied to assess clonality in the context of routine cell line development. Our standard platform is based on the use of the ClonePix FL (Genetix). We thus performed a standard single‐cell cloning procedure involving plating a cell suspension in semi‐solid medium and automated colony picking following strict imaging criteria. Instead of a transfected cell population, however, we used a mixture of two clonally derived cell lines selected for growing at extremely fast and slow rates (referred to as fast grower, or FG, and slow grower, or SG, see “Preparation and detection of non‐clonal colonies during cell line development” in Supplementary Methods). This was performed to mimic the dilution of slow‐growing cells cultured within a population of fast‐growing ones, to ascertain that a mix of two clones growing at highly different speeds could effectively be distinguished using GTC (Figure 4a). Using previously established diagnostic PCR assays designed to identify each of the FG and SG clones, we screened the picked colonies for non‐clonal samples showing both the FG‐ and SG‐specific amplicons (Figure 4b), as opposed to clonal samples showing either the FG‐ or SG‐specific amplicon. Given the very rare occurrence of non‐clonal colonies under standard operating conditions used for cell cloning, we intentionally increased the cell density used to seed semi‐solid medium to decrease the number of colonies that needed to be screened to find non‐clonal ones. Finally, we subjected several clonal and non‐clonal samples to whole‐genome sequencing and GTC in a blinded manner.

Without a priori information about the FG and SG cell lines, GTC correctly identified clonal (i.e., pure) and non‐clonal (i.e., mixed) samples, in line with the PCR screen (Figure 4c). Given that the FG cell line had a shorter doubling time, we expected to obtain a much greater fraction of FG cells compared to SG cells in the case of mixed samples. In line with our expectation, the major clonal fraction of the non‐clonal sample was measured by GTC to comprise 97% of the sample. As a validation, we independently measured clonal proportions using qPCR assays which confirmed the accuracy of GTC (Figure 4d). These results show that GTC provides an agnostic and accurate method that can be used for the assessment of clonality in the context of standard cell line development procedures, even in the very unfavorable case of mixed clones bearing highly different doubling times.

### 3.5 GTC can be applied in cell line development procedures involving successive subcloning steps

**Figure 5:**
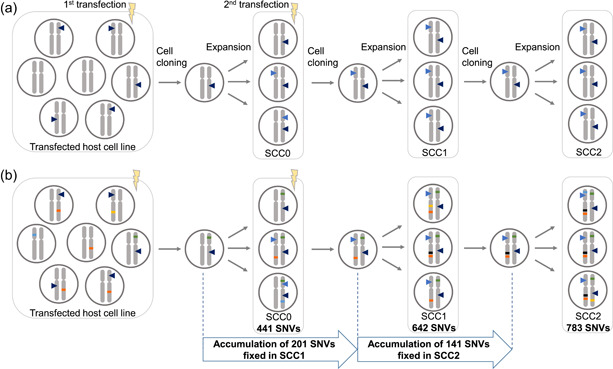
Schematic representation of a multistep process used for commercial cell line development (comprised of two transfections and three rounds of cell cloning) and the genetic evolution of successive subclones. (a) A host cell line is transfected and the transgene (dark blue triangle) randomly integrates into the genome. Upon cell cloning and expansion (SCC0), a second transfection is performed, resulting in additional transgene integration sites (light blue triangle) that increase transprotein production. Two final consecutive rounds of cell cloning are performed to obtain high assurance of clonal derivation for the final cell line (SCC2). (b) Schematic representation of the measured genetic diversity and evolution during successive rounds of single‐cell cloning and expansion. Single nucleotide variants (SNVs) harbored by the progenitor cell selected upon the first transfection (green tick) are fixed in the derived cell line (SCC0). However, new SNVs appear during expansion (orange and blue ticks). The new SNV contained in the next progenitor cell (orange tick) becomes fixed upon expansion of SCC1. Whole‐genome sequencing revealed 207 such SNVs in the SCC1 cell line. The final round of cell cloning selects a progenitor cell that contains an SNV (black tick) that appeared during SCC1 expansion and that becomes fixed in SCC2. Whole‐genome sequencing revealed 150 such SNVs in the SCC2 cell line [Color figure can be viewed at wileyonlinelibrary.com]

Current cell line development procedures can involve multiple transfection and cell cloning steps. For instance, a workflow involving two successive transfection‐and‐cloning rounds may be applied to increase cell line productivity (Figure 5a). Moreover, an additional, final round of cell cloning can be used to ensure the clonal derivation of a cell line designed for therapeutic protein production. In that case, applying GTC to the cell line obtained after the second transfection‐and‐cloning round (SCC1 in Figure 5a) would eliminate the need for a second cloning step, thereby saving time and resources.

The application of GTC, however, requires that sample‐specific SNVs be identified. In the present case of two successive rounds of cloning, the parental population to consider is the cell line obtained after the first transfection‐and‐cloning round (i.e., SCC0 in Figure 5a), not the original host cell line. Indeed, if sample‐specific SNVs are detected by comparison to the host cell line, SNVs identified as fixed in SCC1 may be already fixed in SCC0. Such SNVs will confound the estimation of clonal fractions in the case of failed cell cloning because two potential progenitor cells (from the SCC0 population) will carry the same SNVs.

In such a cell line development protocol containing multiple cloning rounds, the parental population is thus freshly clonally derived and its level of genetic diversity is expected to be low. GTC, however, requires that the parental population contains enough genetic diversity to distinguish potential subclonal populations (Figure S5a,b). Thus, to determine if GTC may be used, we first characterized the genetic diversity in cell populations obtained from successive subcloning steps. Specifically, we sequenced three samples from the same direct lineage (Figure 5a): the cell line obtained after initial transfection and cell cloning (SCC0), the cell line (SCC1) obtained from SCC0 re‐transfection and subcloning and the final cell line (SCC2) obtained from the second consecutive cell cloning. We identified SNVs fixed in each of the three samples by comparing it to the host cell line (Figure S6a). We observed that the number of fixed SNVs steadily increased along this cell lineage, as expected from several rounds of mutation occurrence and fixation during cloning, starting at 441 fixed SNVs in SCC0, and increasing to 642 and 783 fixed SNVs in SCC1 and SCC2, respectively (Figure 5b, and see “Genetic diversity in cell populations obtained from successive subcloning steps” in the Supporting Information and Figure S6b,c).

These results indicated that a newly cloned population subjected to 6–8 weeks of culture already shows a significant genetic diversity. We then asked how rare new SNVs were in the parental population, to make sure that GTC would be able to detect two clonal subpopulations in the case of failed cell cloning (as each of the two progenitor cells that would be selected should carry a specific set of SNVs). Instead of measuring SNV frequency by deep sequencing of SCC0 cells, we reasoned that if new SNVs contained in SCC0 were indeed rare, two different SCC1 cell lines obtained from the same transfection should show very few common, newly fixed SNVs. We thus sequenced an additional SCC1 cell line obtained from the same transfection (see “Verification of model specification: potential presence and influence of shared SNVs” in the Supporting Information and Figure S5c). This SCC1 sister line displayed 634 fixed SNVs, comprised of 425 SNVs found to be fixed in SCCO and 209 newly appeared SNVs. Importantly, all newly fixed SNVs in the sister SCC1 cell line were different from the newly fixed SNVs identified in the original SCC1 cell line (Figure S5d). This demonstrates that if single‐cell cloning performed upon the second transfection had failed (and resulted in the selection of two progenitor cells), each of the two cells would contain hundreds of SNVs that would be absent from the other progenitor cell and that would yield a strong signal for GTC to detect non‐clonality and provide a sensitive measure of clonal fractions. In conclusion, GTC can also be efficiently applied to development protocols involving multiple successive cloning steps where the parental cell line is a recently cloned population.

## 4 DISCUSSION

We report the development of a novel method to assess cell line clonality based on the genome‐wide analysis of SNVs. This genomic test of clonality requires the genome sequencing of the cell line to be tested as well as its parental cell line. We validated the clonal fractions measured by GTC using mixtures of two clonal cell lines and showed that the test is accurate and highly sensitive as it can robustly detect minor clonal fractions of 1%. Interestingly, GTC does not require deep sequencing of the test cell line as it leverages the pooled information gathered over many SNVs. Thus, relatively shallow sequencing (i.e., 25× coverage) yields high‐resolution measures of clonal fractions (i.e., 1% resolution, as fractions of 98% and 99% can be distinguished) and correspondingly tight confidence intervals. GTC is a statistically safe procedure as built‐in calculations verify beforehand that the number and coverage of detected SNVs provide enough statistical power. Specifically, it ensures that a minor clonal fraction of 1% can be detected if it is present (see “Operational threshold for clonal purity” in the Supporting Information).

Cell lines can undergo clonal and genetic evolution, even upon culturing under constant conditions. For instance, two clonal subpopulations that originated from the presence of two progenitor cells at cloning might gradually change in relative abundance over time (as assessed in our experiment using two clones with very different growth rates). We cannot exclude, for instance, that a minor clone having an initially very low growth rate might represent a subthreshold fraction (i.e., <1%) at the time of analysis but might later increase in relative abundance upon subsequent culturing. On the other hand, a clonally derived cell line might spontaneously become heterogeneous upon culturing. To avoid potential issues of genetic evolution, it is thus generally advisable to use GTC on cell lines that have not been subjected to extended culturing times.

The statistical procedure used in GTC, however, is robust against SNVs that would newly appear in the cell population after cloning. Specifically, our method avoids relying on low‐frequency SNVs to infer clonal fractions. This point was illustrated by applying GTC to two clonally derived research cell banks (RCBs). Both RCBs were deemed (clonally) pure (major clonal fractions f = 99.95% and 99.94%). After further development into master cell banks (MCBs), which involved culturing the two cell populations for several additional weeks, they were sequenced and subjected to GTC again: The major clonal fractions estimated by GTC from MCBs (f = 99.96% and 99.85%, respectively) were unchanged compared to what was obtained from the analysis of the RCBs, despite the new SNVs that had inevitably appeared during further culturing.

For cell populations deemed clonally pure, GTC can provide a *p*‐value for clonal‐derivation (“monoclonality”), that is they are derived from a single progenitor cell. Specifically, the confidence for clonal derivation is inversely related to the frequencies of SNVs in the parental cell line. The latter can be measured by deep sequencing of the parental population (e.g. here we used 150× coverage), yielding significant *p*‐values for clonal derivation. Alternatively, an approach combining shallower whole‐genome sequencing of the parental cell line (which is sufficient for the accurate measurement of clonal fractions in the derived cell line, see Methods) followed by deeper, targeted resequencing of a small set of loci might be used to confirm that at least a small subset of SNVs are very rare in the parental cell line and thus provide high assurance of clonality. Importantly, we found, however, that most of the specific SNVs fixed in a clonally derived cell line are at least 10‐fold rarer than what can be measured by standard high‐throughput sequencing methods, indicating that true *p*‐values for clonal derivation are even much smaller than the conservative estimate provided by GTC. Thus, currently, the overall assurance of clonal derivation provided by GTC is effectively conditioned by the sensitivity of GTC to detect minor clonal fractions (conservatively estimated at 1%), rather than by the *p*‐value for clonal derivation as it is comparatively much lower. A future, formal assessment of LOD (limit of detection) may thus bring the overall assurance of clonal derivation beyond 99%.

Current methods used for the routine assessment of clonal derivation during cell line development are usually based on the technical characterization of the cell cloning procedure. When performing limiting dilution, for instance, the probability of selecting two progenitor cells instead of one can be estimated from the density of the cell suspension and appropriate probability calculations. Typically, if one‐third of the wells of the limiting dilution vessel contains cells, then at least one‐third of the cell populations derived from wells containing cells are non‐clonal. Imaging cells during the cloning process can significantly increase the assurance of clonal derivation (see e.g. the review by Chen et al., 2020). It is not devoid of technical difficulties though, ranging from optical performance to automated cell recognition. To date, no method can provide definitive, 100% assurance of clonal derivation. Furthermore, minor changes in a process previously characterized to yield clonal populations with a given probability may also alter statistical confidence, which would not be detected by the experimenter or regulator *a posteriori*. The genetic analysis performed by GTC is the first method that provides direct characterization of a cell line. Here, we also show an example application of GTC in the context of cell line development using an automated imaging and cloning system (Figure 4), which allowed us to detect non‐clonal samples that were not detected by imaging alone.

Furthermore, GTC can be applied to protocols involving multiple cloning steps, such as the subcloning of a recently derived clonal cell line (e.g. when performing successive rounds of transfection and cloning). Indeed, our analysis showed that clonal evolution proceeds at a rate that is sufficient for genetic diversity to reestablish upon weeks of cell culture only. We hypothesize that the underlying origin of these mutations corresponds to the known low mutagenesis background of mismatched bases spontaneously introduced by DNA polymerase during DNA replication, most of which may be neutral from an evolutionary and selection perspective. Indeed, we observed in subclones of recent clonal cell lines that the number of fixed SNVs was proportional to the culturing time preceding cell cloning (Figure 5b and “Genetic diversity in cell populations obtained from successive subcloning steps” in Supplementary Results). Moreover, SNV frequency spectra measured in host cell lines showed exponentially decreasing distributions (Figure S2), in line with the theoretical prediction made for dividing cell populations undergoing a fixed mutation rate. Note also that this type of mutation frequency spectrum, with the rarest mutations belonging to the most frequent mutation type, is ideal for GTC as it ensures with high probability that two random cells in the population contain a majority of different SNVs thereby allowing for highly sensitive detection of two clonal subpopulations in the derived cell line.

In conclusion, GTC can be flexibly implemented in the context of many current cell line development protocols. Given its performance, this direct genetic assessment could eliminate the need for a second round of cell cloning that is often needed to obtain high final assurance of clonal derivation. GTC thus has the potential to allow for faster development timelines. The case of routine cell line development based on a single transfection of a host cell line could particularly benefit from the application of GTC: As the same host cell line is used repeatedly as a parental line, it must be sequenced only once. Subsequently, shallow sequencing of derived cell lines combined with GTC can provide a cost‐effective method to obtain highly characterized cell lines with a high assurance of clonal derivation.

## Supporting information

Supporting information
