# Supplementary material for: Genome‐wide analysis of single nucleotide variants allows for robust and accurate assessment of clonal derivation in cell lines used to produce biologics
Source: Biotechnol Bioeng. 2020 Aug 24;117(12):3628–38. doi: 10.1002/bit.27534 (PMC7818110; doi:10.1002/bit.27534)

Supplementary information

Genome-wide analysis of single nucleotide variants allows for robust and accurate assessment of clonal derivation in cell lines used to produce biologics

Alexandre Kuhn[1,2*], Valérie Le Fourn[2], Igor Fisch[2], Nicolas Mermod[1*]

[1] *Institute of Biotechnology, University of Lausanne, Switzerland*
[2] *Selexis SA, Geneva, Switzerland*

**Corresponding authors:*
Alexandre Kuhn (alexandre.m.kuhn@gmail.com) and Nicolas Mermod (nicolas.mermod@unil.ch)

Content

Material and methods 1
Results 4
Supplementary figure captions 6
Supplementary figures 8

Material and methods

Preparation of in silico mixed clonal samples

We subjected 2 clonally-derived cell lines T1 and T2 (obtained from the transfection of the host cell line HCB-2) to whole genome sequencing. Samples mimicking clonal mixtures with various proportions were generated as follows: Using Picard Tools DownsampleSam (Broad Institute, 2017), we subsampled the sequencing data of T1 and T2 with rates equal to their target clonal fractions in the mixture. We then merged both subsampled datasets using Picard Tools MergeSamFiles (Broad Institute, 2017). This was repeated for every mixed sample, using subsampling rates corresponding to target clonal fractions.

Mixed samples with various proportions of 2 clonally-derived cell lines

We prepared cell suspensions of two clonally-derived cell lines SLX1 and SLX2 (obtained from the same transfection of the host cell line HCB-2) and stained one of them with a green fluorescent dye (CellTracker Green CMFDA Dye, ThermoFisher). Upon measurement of cell densities for both cell suspensions, we prepared mixed samples using the following target ratios (percentage clone 1/percentage clone 2): 0/100, 10/90, 50/50, 90/10, 95/5, 98/2, 99/1, 100/0. We then measured the actual proportions in each sample using a flow cytometer (Guava easyCyte, Luminex). The last 4 samples were subjected to DNA extraction, library preparation and whole-genome sequencing. We then applied GTC to the 4 samples in a blinded manner, i.e. sample labels and flow cytometer measurements were only revealed after genomic analysis was completed.

Preparation and detection of non-clonal colonies during cell line development

Two clonally-derived cell lines showing highly different growth rates were identified from cell clones derived from the transfection and antibiotic selection of the host HCB-1 cell line, termed SG for the slow grower (doubling time: 17 hours) and FG for the fast grower (doubling time: 26 hours). They were used to obtain non-clonal, i.e. mixed colonies containing SG and FG cells. First, we developed 2 diagnostic PCR assays to identify each cell line. In short, we subjected SG and FG cell lines to whole genome sequencing and identified the distinct transgene genomic integration sites present in each of the cell lines using previously described bioinformatic tool (Kostyrko et al., 2017). For each cell line, we then designed a PCR assay specifically targeting the unique chimeric sequence at a genome-transgene junction (i.e. with one primer hybridizing to the CHO genome and the other one to the transgene). The specificity of the 2 diagnostic PCR reactions was verified using genomic DNA from the 2 cell lines as well as appropriate control samples.

To obtain mixed colonies and assess if GTC could detect them, we prepared a mixed cell suspension composed of equal proportions of SG and FG cells that we subjected to single cell cloning using ClonePix (Molecular Devices), a cell colony imaging system widely used for generating clonally-derived populations in cell line development. The cell suspension was plated in semi-solid medium at 150 cell/ml. We used a higher cell density compared to standard operating conditions to increase the probability that the automated colony imaging system would mistakenly pick non-monoclonal colonies. We then picked 75 colonies according to standard imaging criteria (i.e. colony size and roundness). After 3 days of culture, we lysed colonies (CellsDirect, ThermoFisher) and analyzed them using the SG- and FG-specific PCR assays in multiplex (Q5 DNA polymerase, NEB). Finally, amplification products were resolved by 2% agarose gel electrophoresis.

69 samples were successfully genotyped: 27 colonies showed the SG-specific amplicon (200bp) only, 40 colonies showed the FG-specific amplicon (300 bp) only and 2 colonies showed both amplicons (i.e. they were mixed SG/FG samples). We subjected the 2 mixed colonies as well as 1 SG colony and 2 FG colonies to whole genome sequencing and GTC. In addition, we used TaqMan qPCR assays to independently quantify the amount of SG and FG cells in each of the 5 samples (LightCycler 480, Roche). qPCR primers were the same as for the diagnostic PCR reactions, to which appropriate, intervening TaqMan probes (Microsynth AG) were added. The specificity of the assays was verified using genomic DNA from the 2 cell lines as well as appropriate control samples. We ran triplex qPCR reactions comprised of the 2 clone-specific assays and a third assay targeting the reference gene beta-2-microglobulin. The SG and FG content in each sample were quantified using the standard Ct method, normalizing the value of each sample to that of the reference gene, and using a pure SG, or respectively a pure FG sample, as calibrator.

Cell line development involving multiple successive subcloning steps

We investigated the application of GTC in the context of a common cell line development procedure involving 2 transfections and multiple successive cloning steps. In short, the host cell line HCB-2 was transfected and cells were cultured under antibiotic selection for 51 days before they were plated in semi-solid medium (100 cells/ml) for cell cloning using the ClonePix system (Molecular Devices). After 11 days, colonies were picked and expanded (generating “SCC0” cell lines) further for 23 days. Following productivity screening, selected high-producer SCC0 cell lines were subjected to a second transfection (termed supertransfection) to further improve productivity. After 23 days of selection, each supertransfected SCC0 cell line was plated in semi-solid medium (100 cells/ml) and grown for 11 days until colonies were picked and expanded for 31 days (generating “SCC1” cell lines). A second round of subcloning was performed to obtain high assurance of clonality: SCC1 cell lines were plated in semi-solid medium, grown for 10 days, picked and expanded to obtain final cell lines (“SCC2 cell lines”).

A set of 3 cell lines named SCC0, SCC1a and SCC2, derived according to the procedure described above and belonging to the same direct lineage, were subjected to genomic DNA extraction and whole genome sequencing. An additional SCC1 cell line (named SCC1b) obtained from a distinct single cell clone originating from the same supertransfection as SCC1a was also analyzed in parallel.

Whole genome sequencing

Genomic DNA extraction was performed using DNeasy Blood and Tissue Kits (Qiagen). Whole genome sequencing libraries were prepared using TruSeq Nano DNA Library Prep (Illumina) and sequenced on Illumina HiSeq 4000 with read length of 2x150 bp, except for samples of the SLX1/SLX2 clone mixing experiment and the samples SCC0, SCC1a, SCC1b and SCC2 that were sequenced on HiSeq2500 with read length of 2x125 bp. All samples were sequenced on 1 lane of a flowcell (corresponding to an average CHO genome coverage of approximately 25x), except HCB-1 and HCB-2 that were sequenced on 2 and 6 lanes, respectively. Library preparation and sequencing was performed by Fasteris SA (Plan-les-Ouates, Switzerland).

Analysis of sequencing data and detection of SNVs

We aligned sequencing data on the CHO-M reference genome sequence (v4, Selexis) using bwa (Li & Durbin, 2009), and reads were subsequently filtered using SAMtools (Li et al., 2009) following standard practice. We detected sample-specific SNVs by comparing samples with the corresponding host cell line using the Bioconductor/R packages VariantTools (M Lawrence, Degenhardt, & Gentleman, 2019) and gmapR (Barr, Wu, & Lawrence, 2019).

We identified 1-copy regions of the genome as based on read counts as follows: We first counted sequencing reads over 1000 bp-bins genome-wide. Upon correction to account for variation in GC content across bins and removal of regions with low mappability (Derrien et al., 2012), the read count data was normalized to the total sequencing depth of the sample, and the count profiles were smoothened and segmented in order to detect the locations and boundaries of copy number changes. This procedure was implemented using the following Bioconductor/R packages: GenomicAlignments (Michael Lawrence et al., 2013), cn.mops (Klambauer et al., 2012), rtracklayer (Michael Lawrence, Gentleman, & Carey, 2009), HMMcopy (Lai, Ha, & Shah, 2019) and DNAcopy (Seshan & Olshen, 2019). Computations were performed on the computing cluster of the Vital-IT Competence Center (Swiss Institute of Bioinformatics, Lausanne).

Operational threshold for clonal purity

Following the experimental demonstration that GTC can robustly detect a minor clonal fraction of 1%, and since we did not test smaller minor fractions, we empirically define “clonal purity” as a major clonal fraction of at least 99%. To be more conservative when calling purity, however, we use the lower limit of the confidence interval for $\mathbf{f}_{\mathbf{binom}}$ as threshold (instead of $\mathbf{f}_{\mathbf{binom}}$). In other words, a sample is called pure only if the lower limit of the Agresti-Coull confidence interval for $\mathbf{f}_{\mathbf{binom}}$ is > 0.99.

As described above, the major clonal fraction **f** is estimated from the sum of the Binomial variables modeling SNVs from the majority clone. This implies that the accuracy on $\mathbf{f}_{\mathbf{binom}}$ is proportional to the number of SNVs and their coverage. Specifically, if the true probability of observing an alternative allele is 1 (i.e. major clonal fraction is 1), at least 460 trials are needed such that the lower limit of the confidence interval for the fraction is > 0.99. Thus, before calculating $\mathbf{f}_{\mathbf{binom}}$, we verify that $\sum \mathbf{c_d}$ is greater than 460 (which implies that we have the minimal number of SNVs and coverage to confidently call a pure sample). For the sake of illustration and considering a uniform coverage of 13, this implies for instance that at least 36 SNVs (each showing only the variant allele) are necessary to call a sample “clonally pure”.

Results

Verification of model specification: potential presence and influence of shared SNVs

SNVs that are shared by 2 progenitor cells (i.e. in the case of failed cell cloning) can potentially bias the measure of the major clonal fraction (towards 1) because they will appear as fixed in the derived (polyclonal) population. In our application of GTC, we ensured that analyzed SNVs were specific to a given clone by selecting only SNVs with very low frequency in the parental population, hence reducing the probability that 2 randomly drawn cells carry the same SNV. Specifically, we ensured low SNV frequency (< 0.05) in the parental population by performing deep sequencing of the parental cell population.

To experimentally assess the actual number of SNVs that are shared across 2 random progenitor cells, we identified the number of common SNVs present among the fixed SNVs detected in 2 clonally-derived cell lines obtained from the same population. Indeed, fixed SNVs that can be found in both cell lines 2 cell lines correspond to SNVs that were present in both progenitor cells. We investigated the 2 frequent cases used in cell line development, that is cloning from a host cell bank and sub-cloning from a recently cloned population. The latter case is exemplified in this study by the SCC1a and SCC1b samples. The 2 cell lines carried 207 and 209 newly fixed SNVs, none of which was common between the 2 sets (Suppl. Figure 5c and 5d). This indicates that the fraction of shared SNVs among all SNVs that appeared during expansion of SCC0 is very low (< 0.005, i.e. 1/200). The same result held true for 2 other clonally-derived cell lines obtained from a standard host cell population (HCB-2). This indicated that the fraction of SNVs present 2 random cells in these parental populations is very low. Accordingly, this indicates that the set of fixed SNVs may be used to uniquely identify a clonally-derived cell line derived from such parental cell populations.

Additionally, and for the sake of generality, we used numerical simulations to investigate the hypothetical influence of shared SNVs on the performance of GTC. We simulated SNVs from 2 clonal populations according to the parametric model developed for GTC, with parameters set to typical values observed in this study (SNV coverage=13, total number of SNVs=500). We systematically varied the fraction of common SNVs and run GTC on the simulated data to systematically assess their effect on the measure of the major clonal fraction $\mathbf{f_{binom}}$ (Suppl. figure 7). We found that even in the very unfavorable case where 50 SNVs are present in both clones (corresponding to a fraction of shared SNV of 0.1, whereas the experiment above suggested that it is < 0.005), $\mathbf{f_{binom}}$ is only slightly overestimated ($\mathbf{f_{binom}}$ = 0.88 when the true clonal fraction is 0.85). Moreover, the bias decreased with increasing major clonal fraction such that the estimation of highly unbalanced mixtures (where the risk of mistakenly calling a sample as clonally pure is the highest) was only minimally affected ($\mathbf{f_{binom}}$ = 0.992 when the true clonal fraction is 0.99). In conclusion, the robustness of GTC against shared SNVs suggests that the criterion used for SNV selection (frequency in the parental population < 0.05) is very conservative and that it could even be relaxed without compromising the measure of the major clonal fraction. This in turn would allow for shallower sequencing depth of the parental population.

Genetic diversity in cell populations obtained from successive subcloning steps

We studied the case of cell cloning from a recently cloned cell line which occurs in cell line development protocols involving 2 cloning-and-transfection rounds (represented in Figure 5a). We observed that the number of fixed SNVs steadily increased along this cell lineage, as expected from several rounds of mutation occurrence and fixation during cloning, starting at 441 fixed SNVs in SCC0, and increasing to 642 and 783 fixed SNVs in SCC1 and SCC2, respectively (Suppl. figure 6a). Since the 3 cell lines belonged to a single lineage and were subclones of each other, SNVs fixed in SCC0 should be fixed in SCC1 as well. As expected, the vast majority (435) of SNVs fixed in SCC0 were found

among the 642 SNVs fixed in SCC1 (Supppl. figure 6b). We reasoned that the remaining 201 fixed SNVs appeared as de novo mutations during expansion of SCC0, i.e. after selection of the SCC0 progenitor cell (Figure 5b). Indeed, if any of those SNVs were present in this progenitor cell, it would have been found fixed in the SCC0 cell line.

Interestingly, the culturing time that elapsed between isolation of the SCC0 progenitor cell and the next subcloning (that allowed for the additional SNVs fixed in SCC1 to appear) was of 8 weeks. Similarly, for SCC2: the vast majority of SNVs fixed in SCC1 (633 out of 642) were found among the 783 SNVs fixed in SCC2 (Suppl. figure 6c). Correspondingly, the 141 newly fixed SNVs must have appeared during the 6 weeks of culturing between the isolation of the SCC1 progenitor cell and the final subcloning needed to obtain SCC2 (Figure 5b). The number of new SNVs is approximately proportional to the duration of the culture, suggesting that the mutational process occurs at a constant rate.

References

Barr, C., Wu, T., & Lawrence, M. (2019). GmapR: An R interface to the GMAP/GSNAP/GSTRUCT suite (Version R package).

Broad Institute. (2017). *Picard Tools (http://broadinstitute.github.io/picard)*. http://broadinstitute.github.io/picard

Derrien, T., Estellé, J., Marco Sola, S., Knowles, D. G., Raineri, E., Guigó, R., & Ribeca, P. (2012). Fast Computation and Applications of Genome Mappability. *PLoS ONE*, *7*(1). https://doi.org/10.1371/journal.pone.0030377

Klambauer, G., Schwarzbauer, K., Mayr, A., Clevert, D.-A., Mitterecker, A., Bodenhofer, U., & Hochreiter, S. (2012). cn.MOPS : Mixture of Poissons for discovering copy number variations in next-generation sequencing data with a low false discovery rate. *Nucleic Acids Research*, *40*(9), e69. https://doi.org/10.1093/nar/gks003

Kostyrko, K., Neuenschwander, S., Junier, T., Regamey, A., Iseli, C., Schmid-Siegert, E., … Mermod, N. (2017). MAR-Mediated transgene integration into permissive chromatin and increased expression by recombination pathway engineering. *Biotechnology and Bioengineering*, *114*(2), 384-396. https://doi.org/10.1002/bit.26086

Lai, D., Ha, G., & Shah, S. (2019). HMMcopy : Copy number prediction with correction for GC and mappability bias for HTS data (Version R package).

Lawrence, M, Degenhardt, J., & Gentleman, R. (2019). VariantTools : Tools for Exploratory Analysis of Variant Calls (Version R package).

Lawrence, Michael, Gentleman, R., & Carey, V. (2009). rtracklayer : An R package for interfacing with genome browsers. *Bioinformatics (Oxford, England)*, *25*(14), 1841-1842. https://doi.org/10.1093/bioinformatics/btp328

Lawrence, Michael, Huber, W., Pagès, H., Aboyoun, P., Carlson, M., Gentleman, R., … Carey, V. J. (2013). Software for computing and annotating genomic ranges. *PLoS Computational Biology*, *9*(8), e1003118. https://doi.org/10.1371/journal.pcbi.1003118

Li, H., & Durbin, R. (2009). Fast and accurate short read alignment with Burrows-Wheeler transform. *Bioinformatics (Oxford, England)*, *25*(14), 1754-1760. https://doi.org/10.1093/bioinformatics/btp324

Li, H., Handsaker, B., Wysoker, A., Fennell, T., Ruan, J., Homer, N., … 1000 Genome Project Data Processing Subgroup. (2009). The Sequence Alignment/Map format and SAMtools. *Bioinformatics (Oxford, England)*, *25*(16), 2078-2079. https://doi.org/10.1093/bioinformatics/btp352

Seshan, V., & Olshen, A. (2019). DNAcopy : DNA copy number data analysis (Version R package).

Supplementary figure captions

Supplementary figure 1: Parametric modeling of SNVs used in GTC and measure of the major clonal fraction. a: Alternative allele counts at genomic positions carrying SNVs are modeled as a mixture of 2 Poisson distributions. In this example, the upper (red dots) and lower (black dots) component distributions contain SNVs (average coverage 13) contributed by the major (f = 0.9) and minor (1 - f = 0.1) clonal fractions, respectively. The 2 distributions are "separable" as the blue vertical line indicates an allele count value (4) such that SNVs from the major (minor) clone have > 95% (< 5%) chance to show greater alternative allele counts. Exact probability values for a boundary count value of 4 are indicated. b: Distribution of alternative allele counts for SNVs detected in the in silico mixed sample with a clonal ratio of 95/5. The average SNV coverage was 12.4 and the Poisson-based estimate of the major clonal fraction ($\mathbf{f_{pois}}$ = 0.93) was measured by fitting a single Poisson distribution (green dots), with truncation level at 6 (vertical line). c: Minimal clonal fraction (of the majority clone) ensuring separability as a function of SNV coverage. We simulated Poisson counts and analyzed numerically the minimal fraction ensuring separability of the allele counts from the 2 clonal subpopulations. The minimal fraction generally decreases with increasing coverage. The decrease is steeper at low coverage (until approximately 15), such that deeper sequencing has gradually diminishing returns with regards to the improvement in separability. For a coverage of 13, the minimal major clonal fraction ensuring separability is 0.81. d. Alternative allele counts versus coverage for all SNVs detected in the 95/5 in silico mixed sample (same sample as in panel b). The boundary defining SNV identity is represented by blue dots. SNVs assigned to the major clone (i.e. with alternative allele counts greater than the boundary) are used to calculate the Binomial-based estimate of the major clonal fraction $\mathbf{f_{binom}}$ = 0.95 (95% confidence interval = [0.94, 0.95]).

Supplementary figure 2: Allelic frequency spectra for SNVs detected in the host cell line HCB-2 (sequenced at 150x coverage) and a clonally-derived cell line (25x coverage). a: SNVs detected genome-wide in the host cell line. In addition to heterozygous SNPs (with frequency 0.5), there is an abundance of low frequency SNVs. The small proportion of SNVs occurring at a frequency of 1 reflect differences between the reference CHO genome assembly and HCB-2. b: SNVs specific to the clonally-derived cell line (i.e. absent or present at significantly lower frequency in the host line). New SNVs have frequencies centered around 0.5 or 1, corresponding to SNVs fixed in 2-copy and 1-copy regions, respectively. c: Subset of SNVs located in 1-copy regions of HCB-2 show low frequencies. d: Subset of SNVs specific to the clonally-derived cell line and located in 1-copy regions have a frequency of 1, that is they are fixed (as hypothesized in Figure 1b).

Supplementary figure 3: Schematic representation of the composition of the SNV frequency spectrum observed for a sample composed of 2 clonal populations. Top: The clone 1 (blue sequencing reads) contains a fixed SNV (SNV 1, pink ticks) and the clone 2 (green sequencing reads) harbors another fixed SNV (SNV 2, yellow ticks). Bottom: When the 2 clones are mixed, the sequencing data contain reads from both clones, in proportion to the clonal fractions. Importantly, in the mixed sample, none of the 2 SNVs are fixed. SNV 1 has a frequency around 0.2 as reads from clone 1 compose 20% of the mixed sample and SVN 2 has frequency around 0.8 as reads from clone 2 compose 80% of the mixed sample. When analyzing SNVs genome-wide, the SNV frequency spectrum thus shows 2 underlying clusters composed of SNVs that are specific to the minority clone (clone 1, lower cluster) or majority clone (clone 2, upper cluster). The positions of the 2 clusters reflect the clonal proportions in the mixed sample.

Supplementary figure 4: Procedure used for the Genomic Test of Clonality (GTC). The parental (host) cell line and derived cell line are both subjected to whole genome sequencing to identify SNVs that are specific to the derived cell line. GTC then proceeds in 2 consecutive steps. The first step measures clonal fractions. If the derived cell line is deemed clonally pure, a second step estimates the p-value for clonal derivation.

Supplementary figure 5: Application of GTC to the subcloning of a recently cloned cell line and investigation of clonal evolution. We study the case of cell cloning from a recently cloned cell line which occurs in cell line development protocols involving 2 rounds of transfection (represented in Figure 5a). a: If there is no new SNV mutations during expansion of SCC0, 2 progenitor cells accidentally selected during (failed) cell cloning harbor the same SNVs (green tick). These SNVs appear fixed in the derived population and non-monoclonality cannot be detected by GTC. b: When cell expansion is accompanied by mutation and the appearance of new SNVs (green and orange ticks), non-monoclonality can be detected by GTC if new SNVs are sufficiently rare to ensure that 2 progenitor cells have low probability of sharing the same set of SNVs. c: 2 SCC1 sisters cell lines were subjected to whole genome sequencing and SNV analysis. They revealed 642 and 634 fixed SNVs, including respectively 435 and 425 SNVs previously fixed in SCC0. d: Venn diagram showing the sets of SNVs detected in the 2 SCC1 sister cell lines. The common SNVs are SNVs that are fixed in SCC0. All newly fixed SNVs are specific to each of the 2 SCC1 cell lines.

Supplementary figure 6: Analysis of SNVs appearing across multiple rounds of cell cloning and expansion. a: Four cell lines belonging to the same lineage (represented in Figure 5a) were subjected to whole genome sequencing and SNV analysis. The number of SNVs detected in each cell line in comparison to the host cell line (indicated) increases with each round of cell cloning/expansion. b: Venn diagram representing the sets of SNVs fixed in SCC0 (red) and in SCC1 (green) and their overlap. c: Venn diagram representing the sets of SNVs fixed in SCC1 (green) and SCC2 (black) and their overlap.

Supplementary figure 7: Numerical simulation of the effect of shared SNVs on the measure of the major clonal fraction f_{binom}. We used the parametric model described for GTC and numerically simulated 500 SNVs (with average coverage 13) from a mixed population composed of various ratios of 2 clones (85/15, 90/10, 95/5, 82/2 and 99/1). The clonal fraction measured by GTC in the absence of shared SNVs (blue dots) is unbiased and it follows the diagonal (black solid line). Introducing increasing numbers of shared SNVs (25 shared SNVs, green dots or 50 shared SNVs, black dots) results in gradual overestimation of the true clonal fraction. Notably, the bias decreases with increasing major clonal fraction. Each combination of clonal fractions and numbers of shared SNVs was simulated 100 times and error bars show the standard deviations.

Supplementary figures

Supplementary figure 1

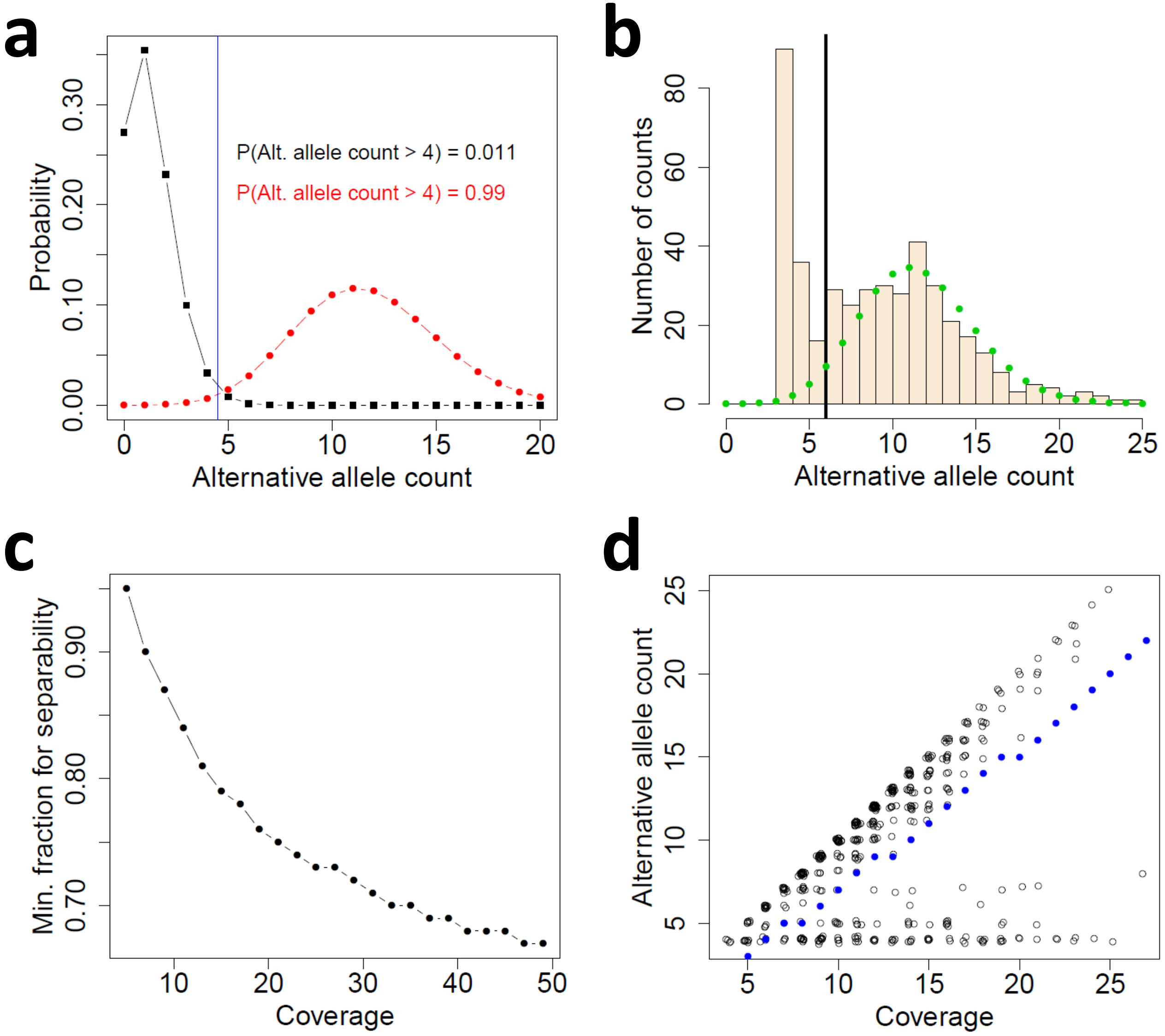

Supplementary figure 2

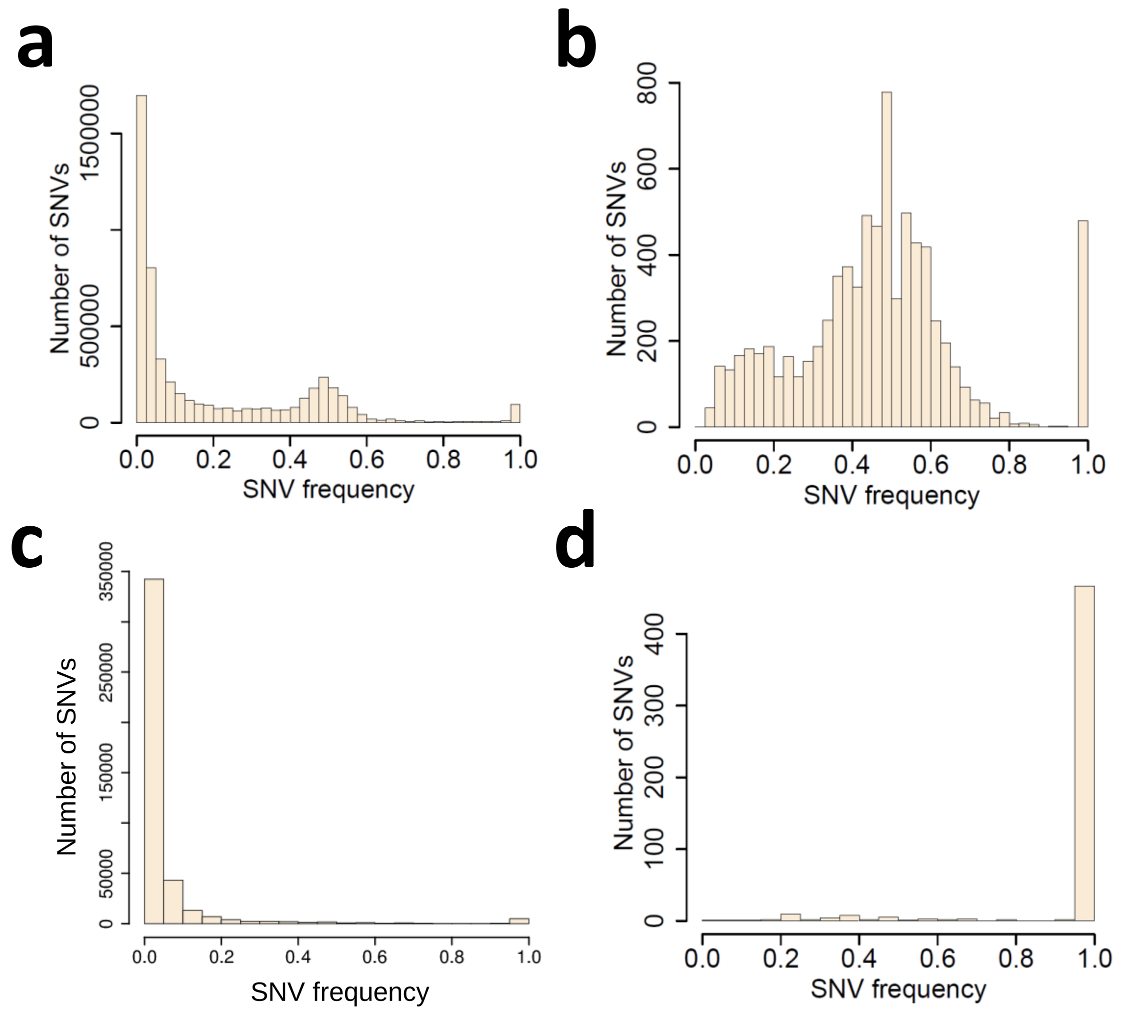

Supplementary figure 3

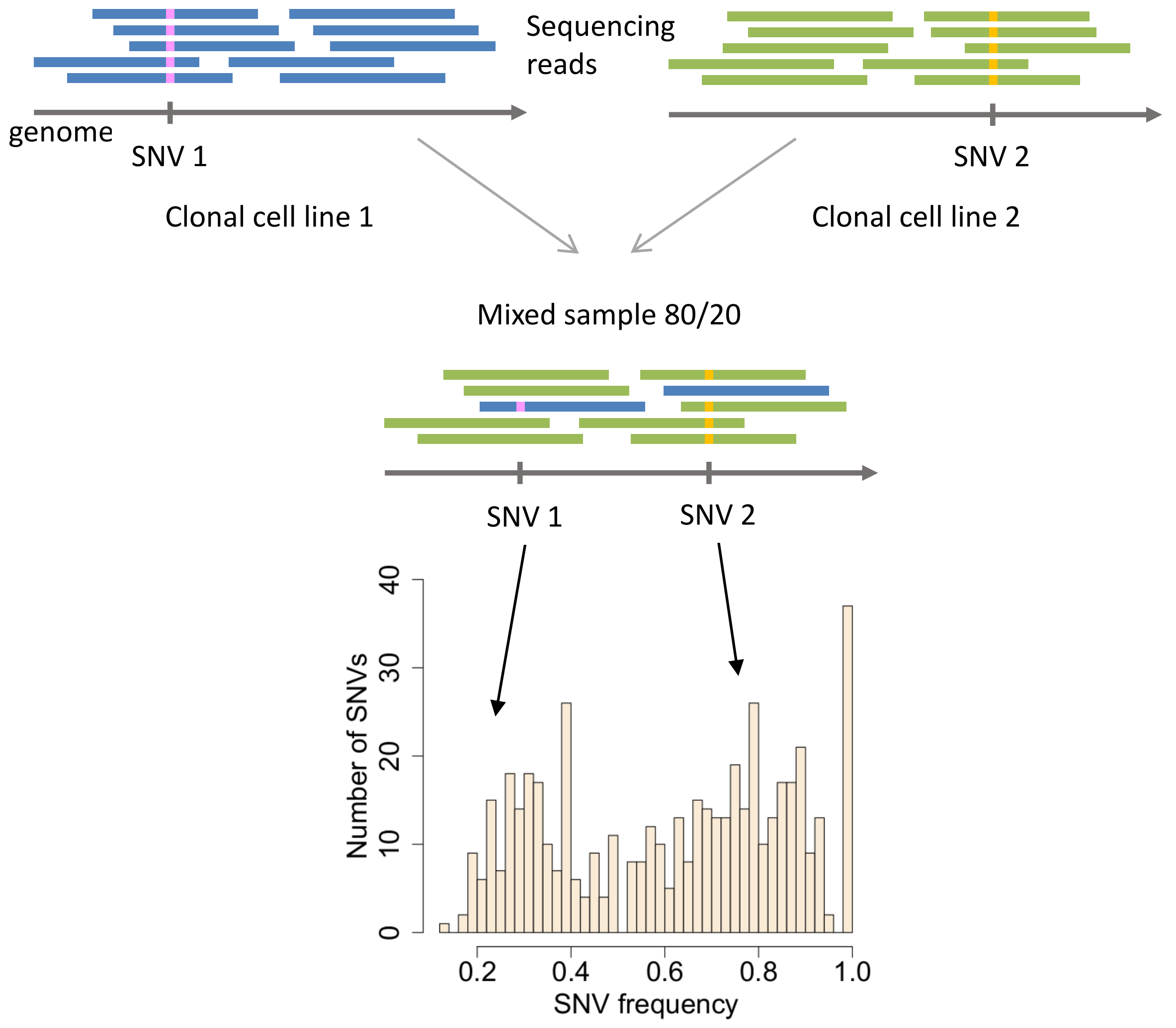

Supplementary figure 4

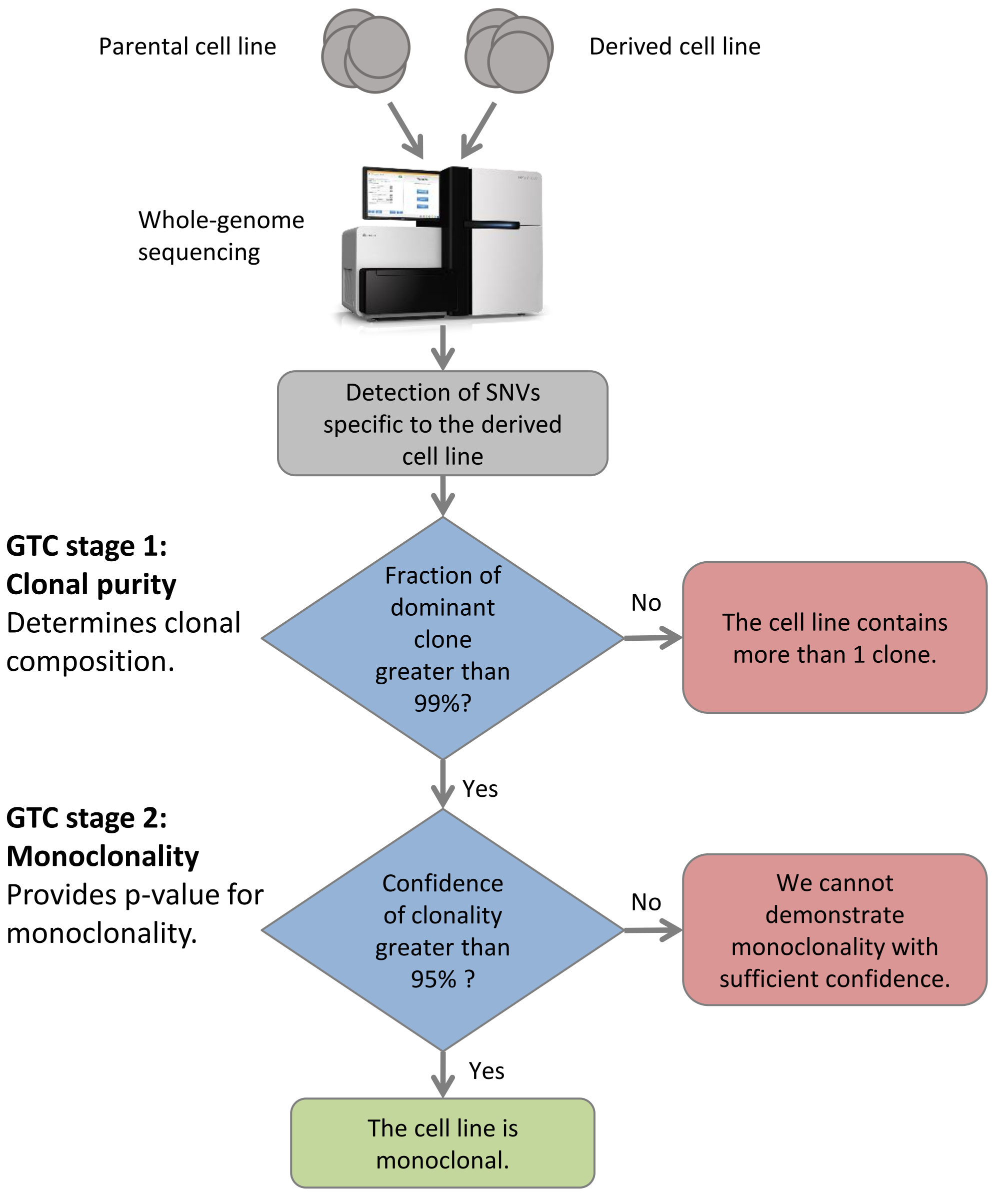

Supplementary figure 5

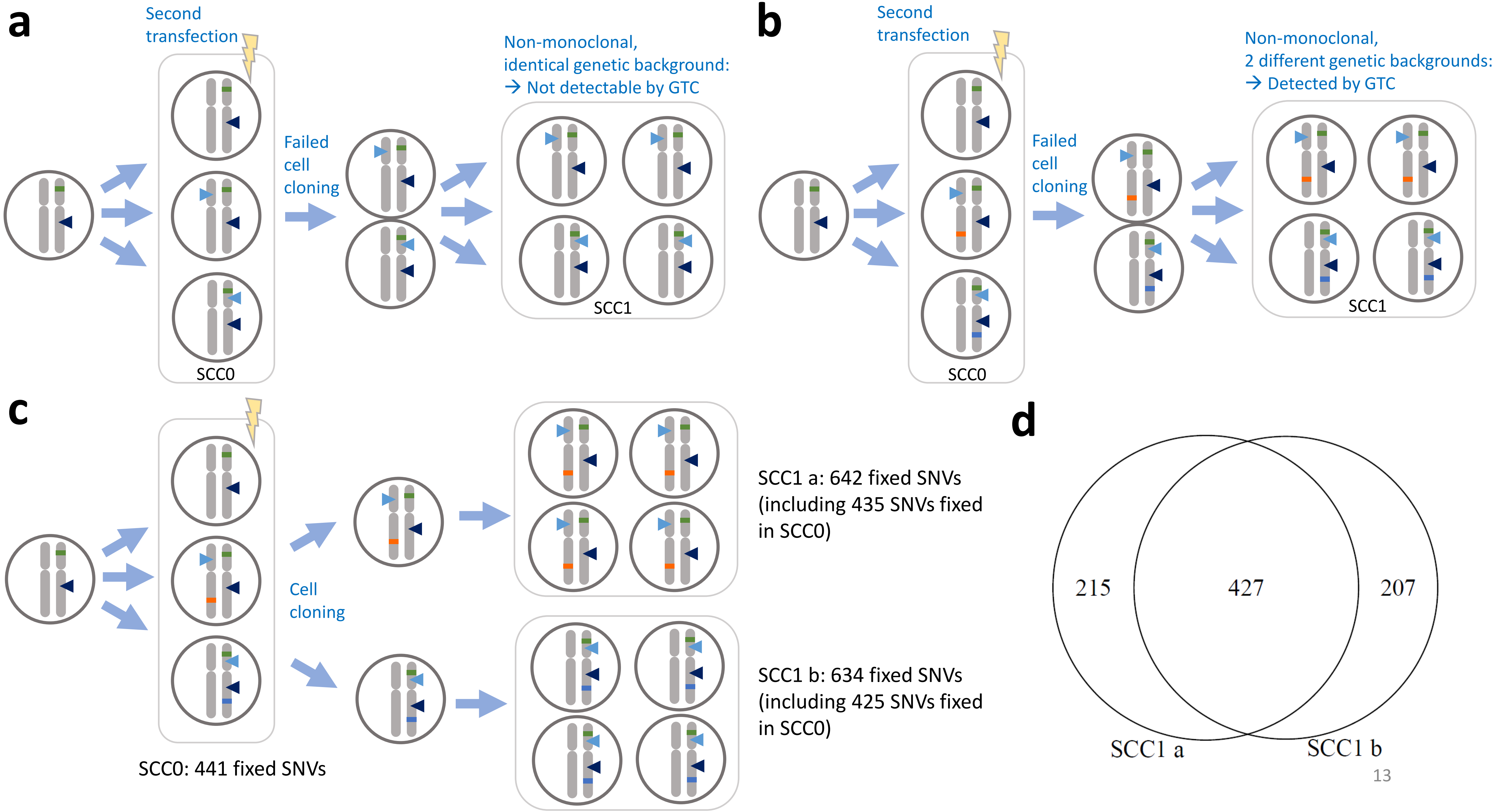

Supplementary figure 6

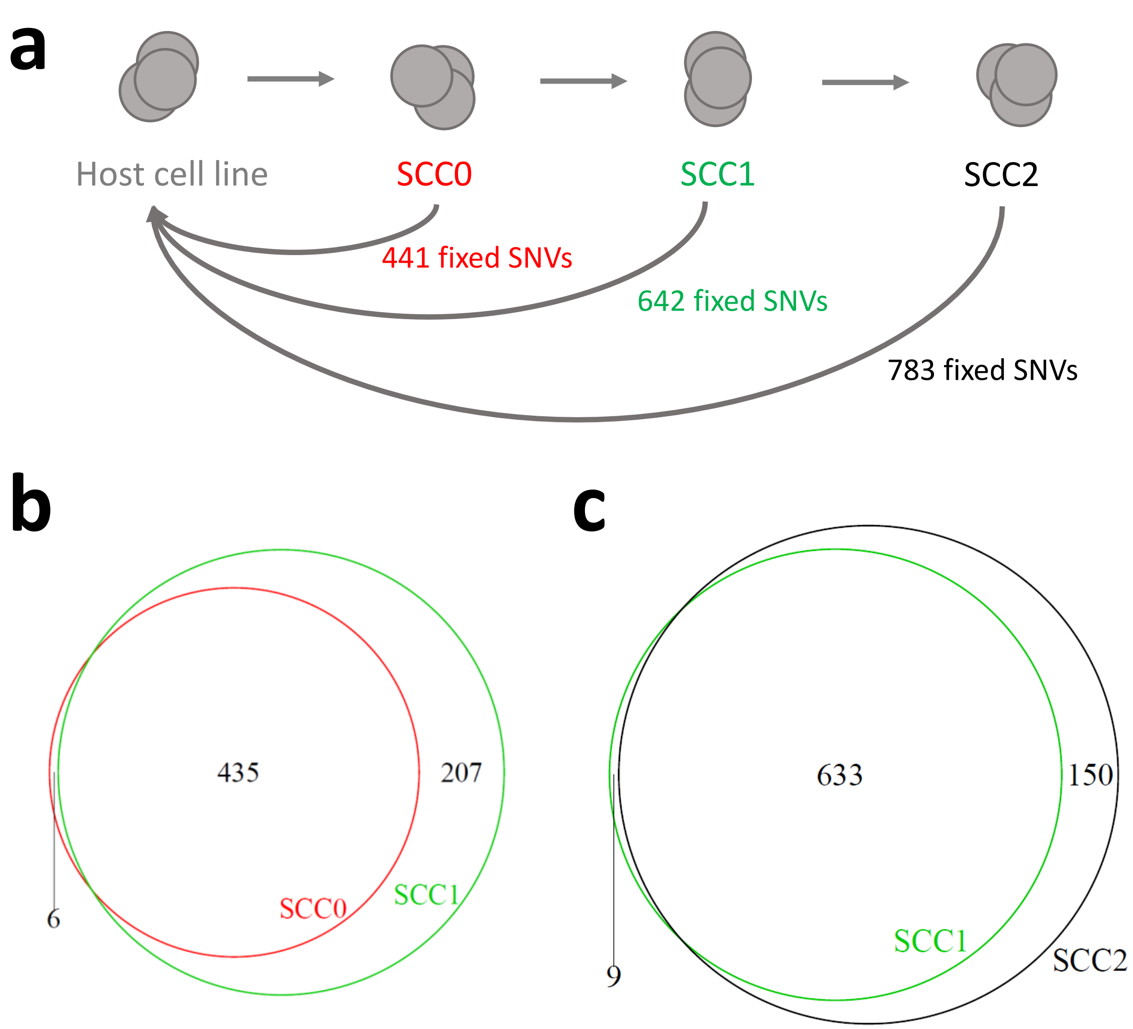

Supplementary figure 7

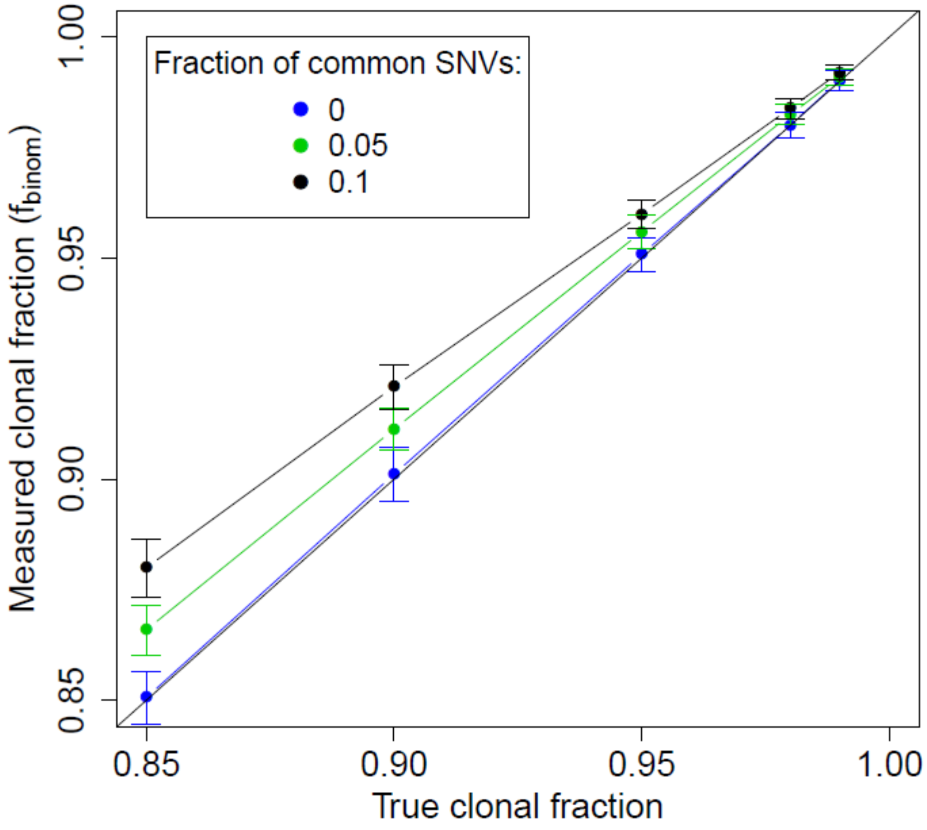

Supplement: Supplementary file 1 — Supporting information [file BIT-117-3628-s001.pdf]
